# Assessment of hybrid population immunity to SARS-CoV-2 following breakthrough infections of distinct SARS-CoV-2 variants by the detection of antibodies to nucleoprotein

**DOI:** 10.1038/s41598-023-45718-8

**Published:** 2023-10-26

**Authors:** Gerco den Hartog, Stijn P. Andeweg, Christina E. Hoeve, Gaby Smits, Bettie Voordouw, Dirk Eggink, Mirjam J. Knol, Robert S. van Binnendijk

**Affiliations:** 1 Centre for Immunology of Infectious Diseases and Vaccines, Centre for Infectious Disease Control, National Institute for Public Health and the Environment, Bilthoven, The Netherlands; 2 Laboratory of Medical Immunology, Radboud Institute for Molecular Life Sciences, Radboudumc, Nijmegen, The Netherlands; 3 Centre for Infectious Diseases, Epidemiology and Surveillance, Centre for Infectious Disease Control, National Institute for Public Health and the Environment, Bilthoven, The Netherlands; 4 Centre for Infectious Diseases Research, Diagnostics and Laboratory Surveillance, Centre for Infectious Disease Control, National Institute for Public Health and the Environment, Bilthoven, The Netherlands

**Keywords:** Adaptive immunity, Applied immunology, Infection, Translational immunology, Vaccines, Diagnostic markers, Translational research, SARS-CoV-2, Viral host response, Infectious diseases, Viral infection

## Abstract

Immunity induced by vaccination and infection, referred to as hybrid immunity, provides better protection against SARS-CoV-2 infections compared to immunity induced by vaccinations alone. To assess the development of hybrid immunity we investigated the induction of Nucleoprotein-specific antibodies in PCR-confirmed infections by Delta or Omicron in vaccinated individuals (n = 520). Eighty-two percent of the participants with a breakthrough infection reached N-seropositivity. N-seropositivity was accompanied by Spike S1 antibody boosting, and independent of vaccination status or virus variant. Following the infection relatively more antibodies to the infecting virus variant were detected. In conclusion, these data show that hybrid immunity through breakthrough infections is hallmarked by Nucleoprotein antibodies and broadening of the Spike antibody repertoire. Exposure to future SARS-CoV-2 variants may therefore continue to maintain and broaden vaccine-induced population immunity.

## Introduction

A large part of the global population has acquired immunity through vaccination, infection or a combination of both i.e. hybrid immunity against SARS-CoV-2 in late 2022^1^. Especially Omicron variants have shown their potential to escape vaccine-induced humoral immunity, resulting in many vaccine breakthrough infections and the development of hybrid immunity^2–7^. Previous infection with Omicron protects against subsequent infections by other Omicron variants, and this protection may be better than hybrid immunity induced by SARS-CoV-2 variants preceding Omicron^8,9^. How Omicron-induced hybrid immunity protects against future variants remains to be seen.

Knowledge about immunological protection induced by vaccines, previous infection, or hybrid immunity is of great importance for COVID-19 intervention policies and further understanding of immunological mechanisms protecting against infectious diseases. Besides that SARS-CoV-2 infection is expected to broaden the immune response as it taps into new antigenic epitopes presented to the immune system, another mechanism explaining increased protection by hybrid immunity is believed to be enhanced mucosal immunity resulting in better local protection against the virus^10^.

Assessment of the development of hybrid immunity in the population requires a clear identification of a passed SARS-CoV-2 infection. Such information has often been obtained from testing registries based on diagnostic SARS-CoV-2 RT-PCR and rapid antigen testing. However, testing behavior and policy varies over time and since April 2022 community testing has been scaled down. Serological testing for virus-induced antibodies could be an alternative to detect SARS-CoV-2 infections and can usually be detected many months after virus exposure.

Immunogenic SARS-CoV-2 proteins that are absent in most vaccines, such as Nucleoprotein (N), can be regarded as a potential tool to identify the development of hybrid immunity through breakthrough infections in a vaccinated population^11^. Both the development of Spike-antibody mediated hybrid immunity and induction of detectable N-specific antibodies require immune activation through replication of SARS-CoV-2 after breakthrough infection. Sufficient immune activation after breakthrough infection may be limited in a proportion of the vaccinated population, due to e.g. presence of vaccine-induced S-specific antibodies that may reduce viral replication, thereby also reducing de novo induced N-specific antibodies following breakthrough infection^12,13^. Identification of breakthrough infections by antibodies to non-vaccine viral antigens would allow for research further elucidating of characteristics of the development of hybrid immunity and also shed light on risk factors for breakthrough infection, e.g. pre-infection antibody levels, virus variants, comorbidities or vaccination status^14,15^.

Next to the induction of N-specific responses, breakthrough infections resulting in immune activation will most likely also boost antibodies towards the spike protein, a mechanism probably casually related to the development of hybrid immunity. Quantifying the additional immune boosting of vaccine targets by an infection in primary and booster vaccination recipients with and without previous infection provides insight into how breakthrough infections support better immunity. Epidemiological studies show a benefit of hybrid immunity over vaccine- or infection-induced immunity^9,16,17^. These findings are substantiated by immunological data showing high levels of antibodies and neutralization in vaccinated persons with a history of infection prior to vaccination^15^. The level and duration of boosting by infections in vaccinated persons is largely unknown, as well as the factors influencing the response.

A major factor for breakthrough infections involves the genetic drift of virus strains, resulting in their potential to escape humoral immunity^18^. Protection against infection from various existing and novel SARS-CoV-2 strains is likely dependent on the ability to acquire B cells recognizing new epitopes. Increased antibody reactivity to a new variant causing relative to antibody reactivity to previous variants could demonstrate the acquisition of such B-cells recognizing new epitopes. Hybrid immunity therefore, may not only boost pre-existing antibody responses but also gain new reactivity to new variants.

Here we assess serological immune response after SARS-CoV-2 breakthrough infection in persons with primary and booster vaccination with and without previous infection. First, we determine the sensitivity of N-antibodies as a tool to identify breakthrough infection. Subsequently, we investigate the boosting of Spike S1-specific responses after breakthrough infection and the influence of time since vaccination and N-seroconversion on the S1-specific antibody levels. The study was performed during the transition period of the Delta variant to Omicron^9^, which provided a unique basis to relate immune activation to the virus strain involved. Therefore, lastly, we investigate the change in the response towards the variant of infection as an indication for the development of broader hybrid immunity.

## Results

### Study population

520 vaccinated persons from the prospective VASCO study with a SARS-CoV-2 infection between October 1st 2021 and February 13th 2022 were enrolled in the study (Table 1). The median age was 55 (IQR 43–64) years and 67% were female. Among the 444 participants of the first round of inclusion in the transitioning period from the Delta to the Omicron variant, 165 (37.2%) had a swab sample which could be retrieved and typed. In addition, 76 cases with an infection between October 1st 2021 and November 15th 2021 were included in the second round of inclusions, categorized as Delta infections based on calendar time. From the first inclusion round 35 (21.2%) were typed by variant-PCR and 130 (78.8%) by whole genome sequencing. Of the breakthrough infections 135 (26.0%) were Delta, 99 (60.0%) were Omicron BA.1 and 7 (4.2%) were BA.2 infections. Age and sex distributions were largely similar between the Delta and Omicron infected individuals. However, different distributions of vaccination status were observed among the Delta and Omicron infected individuals, with individuals experiencing Omicron breakthrough infection more frequently having received a booster vaccination (Table 1). In total, 26 participants (5.0%) were partially vaccinated and 494 (95.0%) completed their primary schedule, of which 236 (47.8%) participants also received one (n = 230, 97.5%) or more (n = 6, 2.5%) booster doses. Samples prior to infection were available with a collection time of 60 days (median, with minimal 3 and maximal 233 days) before the reported positive SARS-CoV-2 test date. Post-infection measurements were taken 22 days (median, at minimal 5 and maximal 75 days) after the reported positive SARS-CoV-2 test date. Of the included individuals, 32 out of 479 (7.5%) had evidence of a previous infection.

**Table 1:** Characteristics of study participants (n = 520).

	Delta	Omicron	Unknown
N (%)	135 (26.0)	106 (20.4)	279 (53.7)
Age group			
18–29	3 (2.2)	9 (8.5)	16 ( 5.7)
30–44	22 (16.3)	23 (21.7)	70 (25.1)
45–59	34 (25.2)	34 (32.1)	89 (31.9)
60–74	71 (52.6)	40 (37.7)	101 (36.2)
75 +	5 (3.7)	0 ( 0.0)	2 ( 0.7)
Unknown	0 (0.0)	0 ( 0.0)	1 ( 0.4)
Sex			
Female	83 (61.5)	65 (61.3)	201 (72.0)
Male	52 (38.5)	41 (38.7)	78 (28.0)
COVID-19 symptom status			
Asymptomatic	19 (14.1)	21 (19.8)	74 (26.5)
Mild symptomatic	35 (25.9)	27 (25.5)	73 (26.2)
Symptomatic	78 (57.8)	56 (52.8)	129 (46.2)
Unknown	3 (2.2)	2 ( 1.9)	3 (1.1)
Number of pre infection samples			
0	7 (5.2)	10 ( 9.4)	31 (11.1)
1	106 (78.5)	82 (77.4)	182 (65.2)
2	13 (9.6)	10 (9.4)	55 (19.7)
3	9 (6.7)	4 (3.8)	11 ( 3.9)
Interval between pre infection sample and SARS-CoV-2 positive test in days mean (SD)	109.93 (48.60)	62.44 (39.28)	77.02 (52.99)
Interval between post infection sample and SARS-CoV-2 positive test in days mean (SD)	26.22 (12.46)	24.90 (9.73)	20.83 (8.64)
Evidence of previous infection			
Yes	6 (4.2)	2 (1.9)	24 ( 8.6)
No	125 (92.6)	95 (89.6)	227 (81.4)
Unknown	4 (3.0)	9 (8.5)	28 (10.0)
Vaccination status (at post infection measurement)			
Partially	15 (11.1)	3 ( 2.8)	8 ( 2.9)
Full	117 (86.7)	29 (27.4)	112 (40.1)
Booster	3 (2.2)	74 (69.8)	159 (57.0)
Vaccine type primary series (at post infection measurement)			
AstraZeneca (Vaxzevria)	39 (32.5)	33 (32.0)	75 (27.8)
BioNTech/ Pfizer (Comirnaty)	71 (59.2)	39 (37.9)	115 (42.6)
Janssen	5 (4.2)	11 (10.7)	27 (10.0)
Moderna (Spikevax)	5 (4.2)	20 (19.4)	53 (19.6)
Vaccine type booster series (at post infection measurement)			
BioNTech/ Pfizer (Comirnaty)	0 (0.0)	36 (48.6)	87 (54.7)
Moderna (Spikevax)	3 (100.0)	38 (51.4)	65 (40.9)
Unknown	0 (0.0)	0 ( 0.0)	7 ( 4.4)

### Serological response post-infection

**Figure 1:**
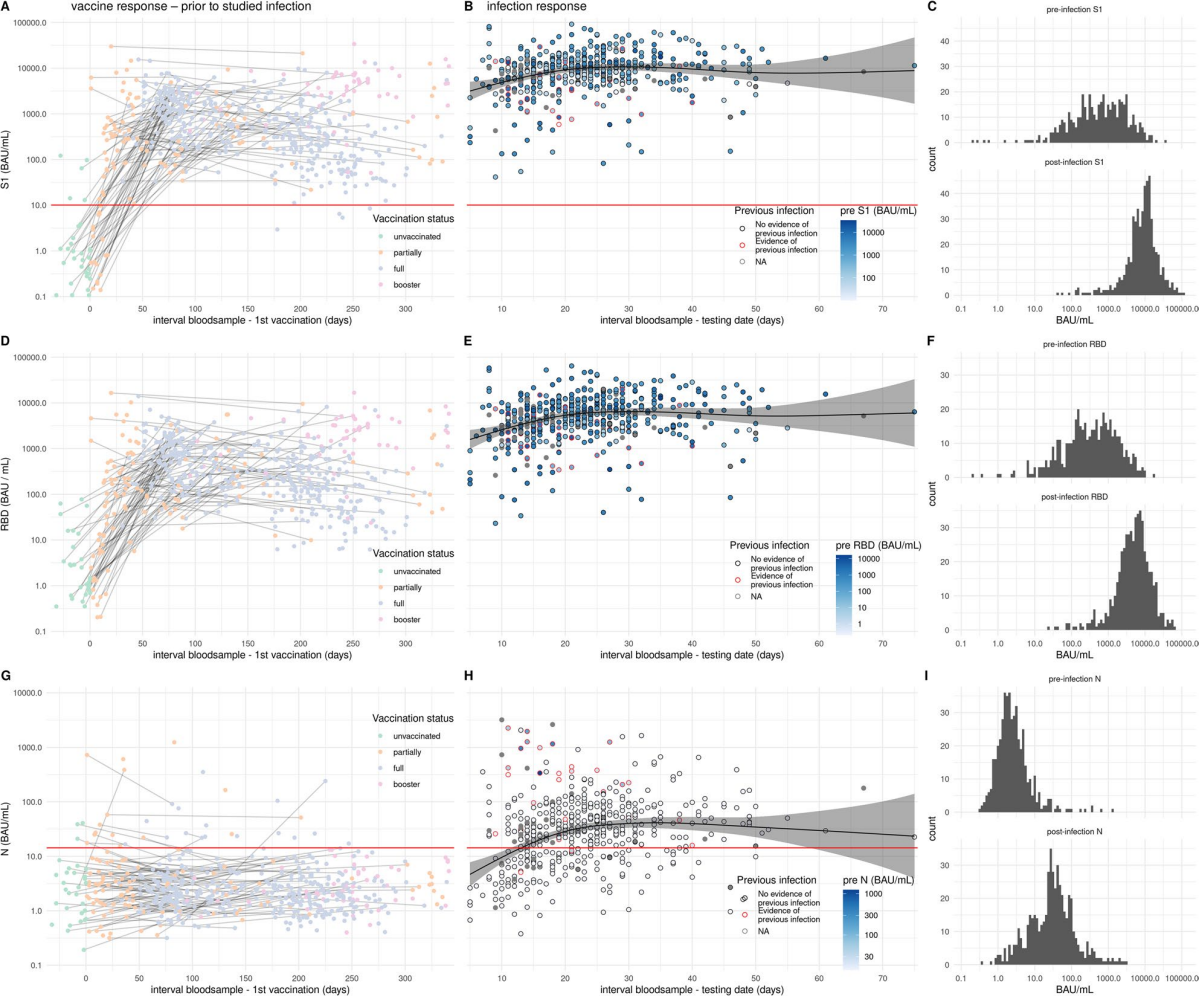
Antibody levels following vaccination and breakthrough infection. (**A**, **D**, **G**) pre-infection antibody concentrations by time since first vaccination for N-, S1, and RBD-specific IgG, respectively (n = 598). Colors indicate the vaccination status at the time of blood collection. Measurements from the same individual are connected (gray line). (**B**, **E**, **H**) Post-breakthrough infection antibody concentrations by time since positive test for N-, S1-, and RBD-specific IgG, respectively (n = 520). Circle colors indicate the history of previous infection (see methods) and circles are filled by pre-infection N, S1, or RBD concentration. Absent pre-infection sample is indicated in grey. Black line shows the estimated mean serological response in not previously infected. Shaded areas represent 95% confidence envelopes. Red horizontal line indicates the seropositivity threshold for N (14.3 BAU/mL) and S1 (10.1 BAU/mL). (**C**, **F**, **I**) Histograms of the pre-infection and post-infection concentrations for N-, S1-, and RBD-specific IgG, respectively.

Participants were enrolled in the study following reporting an infection provided the individual was vaccinated at least once. Of the enrolled participants, blood samples available prior to the infection were analyzed for antibodies to Spike S1, RBD and Nucleoprotein. Pre-infection samples were collected irrespective of the date of vaccination so between different samples non or several vaccination doses could have been administered (left panels Fig. 1). After vaccination an antibody response is observed to spike S1 and its subdomain RBD (Fig. 1A, D). As expected, N-specific antibody responses were not induced by vaccination (Fig. 1G). Of the previously infected participants 26 (81.2%) had N-specific IgG antibodies in their most recent pre-infection serological measurement and 6 (18.8%) participants reported a previous infection without showing N-specific antibodies in the pre-infection serum (Fig. 1G). After confirmed breakthrough infection, parental N-, S1- and RBD-specific antibody concentrations increased with time since positive test and saturated after 4–5 weeks (Fig. 1B and C, E and F, H and I). In previously uninfected individuals the geometric mean antibody concentration (GMC) for N reached 35.7 BAU/mL at 4 weeks after infection (Fig. S1). In persons with a history of infection prior to breakthrough infection the concentration of N-specific antibodies at 4 weeks was 246.7 BAU/mL (Fig. S1). Following breakthrough infection the levels of S1 were boosted reaching a GMC of 10,829.9 BAU/mL after 5 weeks, and in the second week after infection concentrations of 4000–6000 BAU/mL were already observed (Figs. 1B, S1). Individuals with a positive N-specific IgG pre-measurement had an equal geometric mean S1 IgG concentration after infection compared with individuals without previous infection (Figs. 1B, S1).

### N seropositivity following breakthrough infection

**Figure 2:**
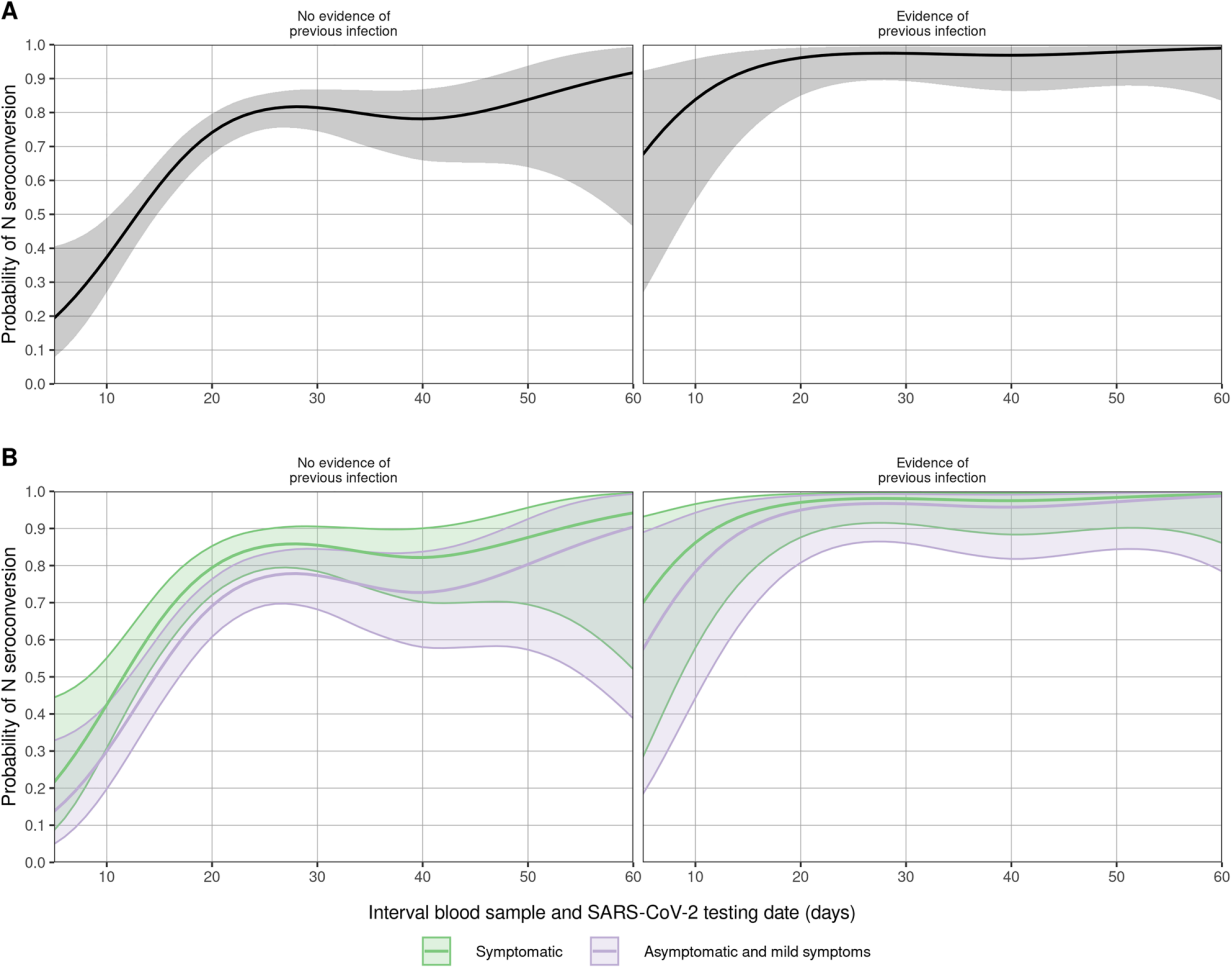
Estimated probability of N-seropositivity by time since positive test. (**A**) Estimates of the probability of N-seropositivity as a function of time since positive test and history of previous infection (n = 479). Shaded areas represent 95% confidence intervals/envelopes. (**B**) Estimates of the probability of N-seropositivity as a function of time since positive test, history of previous infection and COVID-19 symptom status (n = 474). Shaded areas represent 95% confidence intervals/envelopes.

In individuals without a history of previous infection, the probability of N-seropositivity was 54% (95% CI 46–62) and 82% (95% CI 75–86) at two weeks and four weeks after positive test, respectively (Fig. 2A, left panel). In persons with a previous infection 30 out of 32 (94%) were N-seropositive after the current infection (Fig. 2A, right panel. In these persons N seropositivity was mostly already achieved in the second week after breakthrough infection (8 out of 9). Persons reporting symptoms did not show a significant higher proportion of N positivity compared to persons considered mild symptomatic or asymptomatic (*p* = 0.12, Fig. 2B). Neither did seropositivity differ between Delta and Omicron infections (*p* = 0.3) nor vaccination history (vaccination status: partially, full and booster, *p* = 0.2, last vaccine brand used, *p* = 0.9, time since vaccination, *p* = 0.09).

### S1 antibody levels as a function of time since infection and vaccination

**Figure 3:**
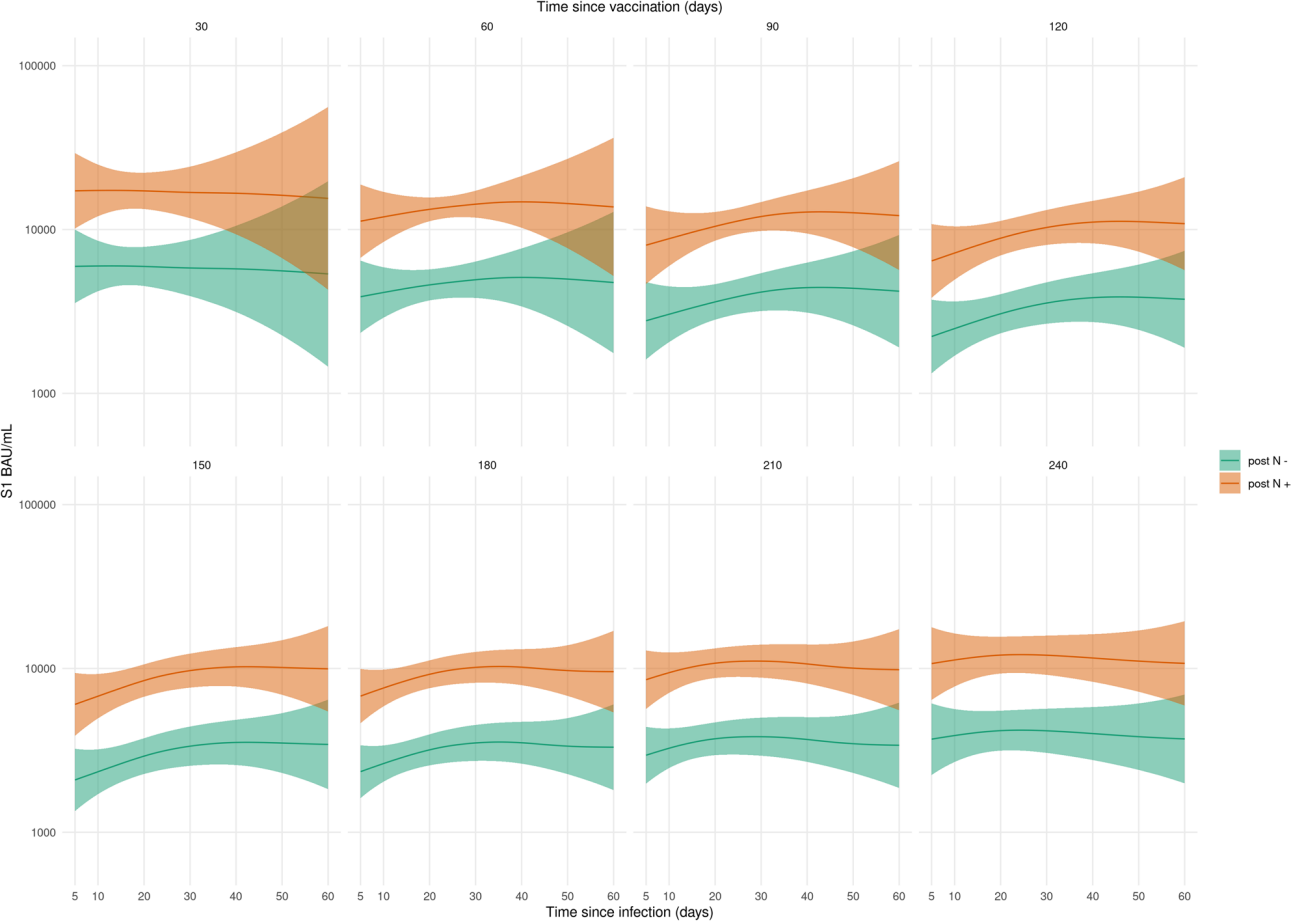
Estimates of the mean S1 antibody levels as a function of time since infection and vaccination in not previously infected (n = 447). Panels show the different time since vaccination (30 days intervals) and *x*-axis the time since breakthrough infection. Orange and green indicate the persons with and without N-specific antibodies following breakthrough infection. Shaded areas represent 95% confidence intervals/envelopes. RBD estimates are shown in Fig. S2.

We found a decrease in Spike S1 levels with increasing time since vaccination. Upon breakthrough infection, increasing time between the last vaccination and breakthrough infection was associated with a faster increase and higher levels of Spike S1 antibodies (*p* < 0.001, Fig. 3). Individuals with measurable N-specific antibodies post-breakthrough infection showed higher levels of antibodies to Spike S1 (*p* < 0.001, Fig. 3) compared to individuals who failed to seroconvert for N. Similar results were found for antibodies to RBD compared to Spike S1 (Fig. S2). As expected, the N-specific antibody response develops independent from vaccination (*p* = 0.22) and is only affected by time since infection (*p* < 0.001, Fig. 1H). Individuals with N seropositivity prior to breakthrough infection had lower levels of Spike S1 antibodies after infection (Fig. S1B, *p* < 0.001).

The level of N and S1-specific IgG antibodies and the duration to reach peak levels was also independent of the virus variant (*p* = 0.88 and *p* = 0.10, respectively).

### Response towards the SARS-CoV-2 variant of infection

**Figure 4:**
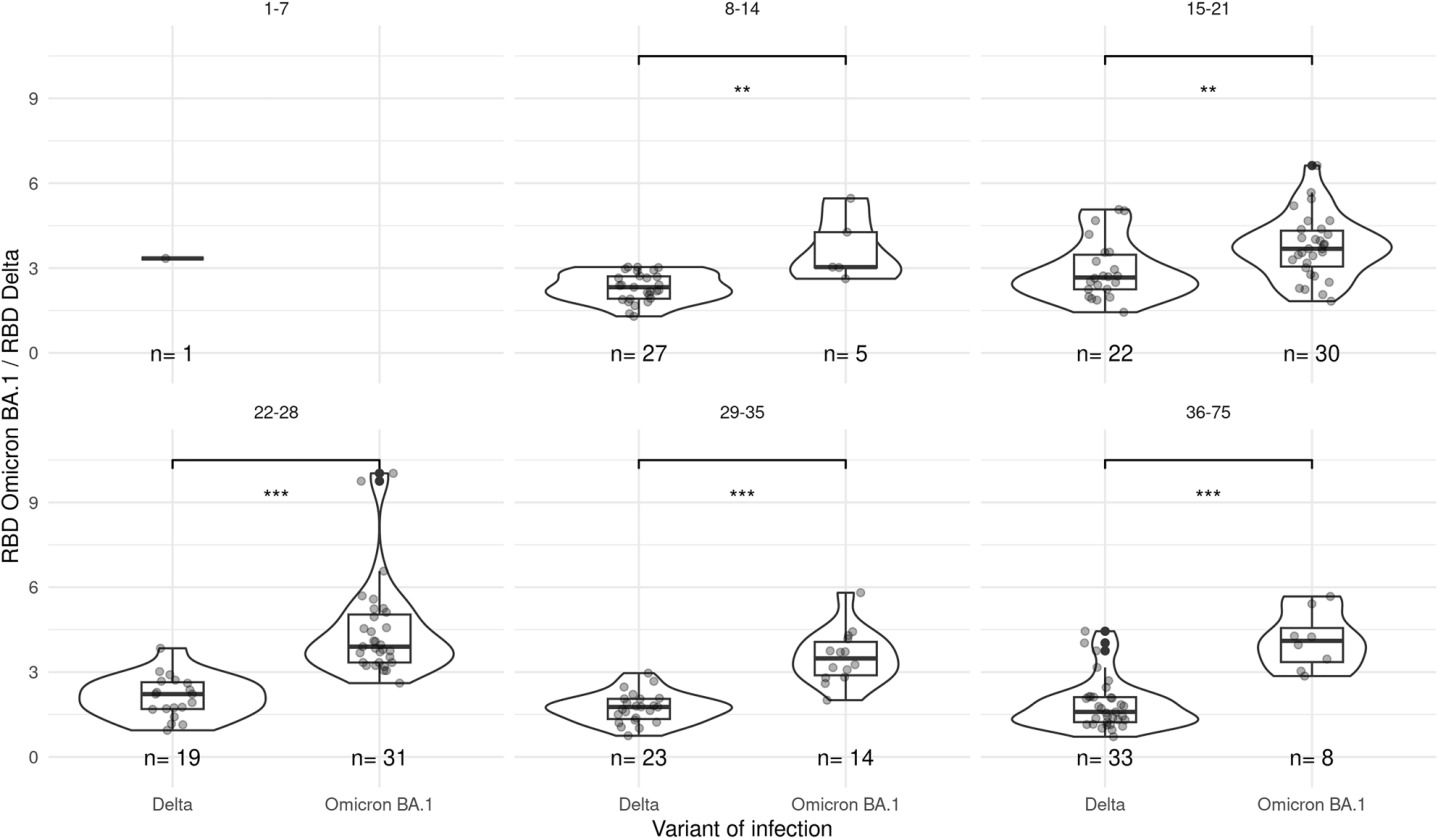
Ratio of the RBD Omicron BA.1 over RBD Delta serological response for Delta and Omicron BA.1 infections in not previously infected individuals. Different subplots indicate time since infection in days. See Fig. S3 for additional antigenic target ratio results. P-value is indicated with ***for < 0.001, **for < 0.01, *for < 0.05 and not significant (NS).

To investigate whether novel type-specific antibodies were induced by Omicron infections we compared the ratio of antibodies to Delta and Omicron BA.1 RBD for persons with a Delta and Omicron breakthrough infection. Ratios were used instead of comparing antibody levels, as both RBD variants will bind a significant proportion of cross-reactive antibodies after breakthrough infection, which cannot be directly compared, as both are scaled according to their own allocated arbitrary unitage. If no variant-specific antibodies are induced following breakthrough infection the ratio between Omicron BA.1 and Delta antibodies should be the same between the groups infected with the Delta and the Omicron variant, with differences in the ratios indicating variant-specific antibodies being induced. Compared with Delta breakthrough infections, Omicron BA.1 breakthrough infections resulted in a higher ratio of Omicron BA.1 over Delta antibodies, indicating the generation of Omicron-specific antibodies (Fig. 4). A similar pattern in ratio towards variant of infection is observed for RBD Omicron BA.1 over parental and RBD parental over Delta (Fig. S3A,B). However, S1 Omicron BA.1 over WT did not differ by variant (Fig. S3C).

## Discussion

The aim of this study was to investigate the humoral immune response following infection in vaccinated persons and how this relates to two different SARS-CoV-2 virus variants responsible for the breakthrough infections, i.e. Delta and Omicron. We show that SARS-CoV-2 breakthrough infections can be identified by N-specific IgG antibodies and boosting of vaccine-induced immunity by infection, leading to hybrid immunity. We show that up to 82% of the individuals that experienced their first infection with SARS-CoV-2 after vaccination developed antibodies to N of SARS-CoV-2, regardless of the virus variant and independent from COVID-19 vaccination. Following breakthrough infection, N-seroconversion was associated with increased S1 antibody levels. N seroconversion might therefore be a more reliable proxy for the development of hybrid immunity rather than a positive PCR or antigen test only confirming breakthrough infection.

To date, very few studies have systematically investigated N-specific seroconversion as a useful marker for breakthrough infection, let alone to relate this to the induction of hybrid immunity^19^. In a recent population serosurvey among individuals without a history of COVID-19 vaccination, 79% of the participants that had reported a PCR-confirmed infection and clinical symptoms were N seropositive between 2 and 6 weeks after infection. For PCR-positive individuals that did not report symptoms, this was 67%^11^. Despite vaccine-induced immunity, the sensitivity to N in the current study was found to be similar to what we and others have found in unvaccinated populations^11,20^. Our estimates of N seropositivity after breakthrough infection were a little lower compared to a recent study by Mizoue et al.^19^ that reported N seropositivity ranging from 78% up to 97% for those infected 4–5 months after vaccination, but not as low as 26% (95% CI 11–49) as reported by Allen et al., the latter of which concerned a small number of investigated persons and with no clear documented timeline of infection^13^. The differences between these two studies and ours could be related to the timing between infection and antibody measurement. The sensitivity of the detection of breakthrough infections by N-specific antibodies is dependent on a minimum time since infection estimated to be about 3 weeks. On the other hand, waning of N-specific antibodies in individuals has been noticed to occur in infected (nonvaccinated) persons, resulting in partial loss in N seropositivity within 5–6 months^11^. Whether a similar waning occurs after breakthrough infection needs to be determined, whilst such an assessment may be hampered by new and consecutive breakthrough infection events. Still, our data support the identification of vaccine-breakthrough infection by the detection of N-specific antibodies applicable within a timeframe of at least 6 months after breakthrough infection. That Nucleoprotein antibodies are induced at similar rates for Delta and Omicron SARS-CoV-2 is likely because the Nucleoprotein is conserved and doesn’t show the many mutations observed for the Spike protein. In addition, S1 antibodies are reported to persist longer than N antibodies, resulting in extended duration of hybrid immunity.

It is interesting to note that a small subset of participants with RT-PCR/rapid antigen-confirmed breakthrough infection (6.7%) had most likely experienced earlier infection prior to this study. This group of participants was characterized by a rapid onset of N antibodies, almost complete (93.8%) N-seropositivity following the breakthrough infection and also reaching much higher levels, indicative for a secondary response to the N protein.

In vitro assays have shown largely reduced neutralization of Omicron variants by pre-Omicron convalescent sera and by sera of individuals vaccinated by monovalent vaccines^3,4,6,18^. In addition, epidemiological studies have shown immune escape by the Delta and Omicron variants^2,21^. Apart from immune escape, a few reports suggest that the vaccines used against SARS-CoV-2 provide a limited degree of mucosal immunity^22,23^. Limited mucosal immunity after primary and booster vaccination may allow a more replication of the virus after exposure, leading to immune activation and the generation of antibodies to the internal Nucleoprotein of the virus. Following such initial replication, the pre-existing immunity by B cell-derived antibodies or memory T cells may also enhance the activation of the immune system resulting in not only boosting of Spike-specific antibodies (hybrid immunity), but also de novo antibodies to other viral targets, while in parallel also improving mucosal immunity because of a first contact with infectious virus at the mucosal site^23,24^. If that indeed happens, hybrid immunity is not only characterized by increased antibody levels and enhanced mucosal immunity, but also by broadened immune responses^25,26^. That this broadening of immunity may occur is indicated by our finding of relatively higher Omicron-specific RBD antibodies in individuals experiencing Omicron breakthrough infections compared to persons with a Delta breakthrough infection. This broadening may continue to occur with subsequent exposures to other variants of the virus. To better assess the development of de novo B cell reactivity, future studies with longer follow-up periods and type-specific virus neutralization assays are needed.

There are some limitations to our study. First, infections not directly adjacent to a retrospective antibody measurement to determine the infection, may be missed, e.g. due to antibody waning as described for N^11^. Therefore, this leaves the possibility for an earlier infection to have occurred unnoticed. Secondly, variant of infection changes with calendar time like vaccinations were administered at given time periods resulting in a correlation between protection by vaccination and the virus variant causing the breakthrough infection. Also, previous infection differs between the Delta and Omicron variants. This leads to differences in vaccination and previous infection status between Delta and Omicron infections, where Omicron cases more often had received their COVID-19 booster vaccination.

In conclusion, protection against future variants by antibodies as determined by antibody concentrations, overlap in antibody-binding epitopes and affinity to the targets. Here we showed that breakthrough infection results in de novo responses to non-vaccine targets, resulting in detectable N-specific IgG antibodies in 82% of the cases. We propose that the observed association of N seroconversion with stronger boosting of S1 antibodies makes the N response a better predictor for the development of hybrid immunity than a positive PCR test, since positive tests in some cases are accompanied with a weak humoral immune response. Although breakthrough infections by distinct virus variants are equally detected by the induction of N antibodies, the breakthrough infections also result in variant-specific antibody levels during the development of hybrid immunity. The generation of de novo responses, the boosting of vaccine-target antibody levels, and broadening of humoral immunity by breakthrough infections likely enhances immunity to current Omicron and future variants.

## Materials and methods

### Study design and population

The VAccine Study COvid-19 (VASCO) is an ongoing prospective cohort study into effectiveness of COVID-19 vaccination in the Netherlands, in which information is collected through regular questionnaires and fingerpick samples for serology were taken every six months^14^. From this study, vaccinated participants with a reported SARS-CoV-2 infection between December 1st 2021 and February 13th 2022 (circulating Delta or Omicron BA.1/2 variant infections) were asked to donate an additional fingerpick blood sample in 1–8 weeks after infection, with outliers up to 11 weeks. After the first round of inclusions, the study was extended with vaccinated individuals with an infection between 1 October up to 15 November 2021 (assumed to be Delta infections, as this was the only variant circulating in the Netherlands at that time^27^) and from which a serum sample was available between 3 and 7 weeks after infection, as Delta infections with a longer interval between infection and blood sample appeared underrepresented in the primary selection.

Data on symptoms were collected directly after a positive test and one month after this positive test. Participants reporting fever, dyspnea, muscle ache, extreme tiredness, general malaise, painful respiration, joint pain, diarrhea, or stomach ache were regarded as COVID-19 symptomatic as these symptoms relate to a systemic infection. Participants with a runny nose, sore throat, anosmia/ageusia, headache, coughing or without symptoms were considered mild symptomatic or asymptomatic as an indication of non-systemic infection.

The VASCO study is conducted in accordance with all relevant guidelines and regulations. The study protocol was approved by the independent Medical Ethics Committee of the Stichting Beoordeling Ethiek Biomedisch Onderzoek (BEBO), Assen, the Netherlands (NL76815.056.21). All participants provided written informed consent.

### Variant detection

Positive national community testing SARS-CoV-2 specimens from participants were collected and variant detection was performed by whole genome sequencing and variant-PCR (S gene target failure), as previously described^9,21^. Sequences obtained in this study are available on GISAID.org (accession IDs provided in Tabel S1).

### Antibody measurements

Nucleoprotein (N)-, Spike S1 (S1)-, and Receptor binding domain (RBD)-specific IgG was measured after breakthrough infection, referred to as post-infection measurement, and in all samples available prior to the breakthrough infection, referred to as pre-infection measurement. Antibodies to antigenic targets N, S1 and RBD of the parental strain, RBD of the Delta variant and RBD and S1 of the Omicron BA.1 variant were detected using a fluorescent bead-based assay, as described previously^18,28,29^. Briefly, samples were diluted (1:400 and 1:10,000) in SM01 (Surmodics, USA) supplemented with 2% FCS and added to the bead mixture. Sample bead mixtures were incubated while shaking in the dark at room temperature. Following washing (3 × PBS) PE-conjugated goat anti human IgG (1:400) was added and incubated for 30 min as above. After washing, samples were acquired on a FlexMap 3D (Luminex) and interpolated using pooled sera calibrated against the international reference (NIBSC, 20/136) and expressed in international units BAU/mL.

The threshold for seropositivity to N (14.3 BAU/mL, reference^11^) and Spike S1 (10.1 BAU/mL, reference^29^) were determined by receiver operator characteristics analysis and mixed modeling using pre-pandemic negative control samples and a heterogeneous mix of samples of PCR-confirmed cases with varying severity (asymptomatic individuals, moderately-ill cases and hospitalized patients)^28,29^.

### Vaccination status and evidence of previous infection

Vaccination status was determined at the date of blood collection. If the self-reported date of blood collection was missing, the received date of the blood sample minus two days (median difference between date of blood collection and date received in non-missing) was used. Partial primary vaccination was defined as having received one dose of Comirnaty, Spikevax or Vaxzevria before date of blood collection, or two doses of Comirnaty, Spikevax or Vaxzevria less than 14 days before this date. Full vaccination is defined as having received two doses of Comirnaty, Spikevax or Vaxzevria at least 14 days before date of blood collection, one dose of Jcovden at least 28 days before this date, or a booster dose less than 7 days before this date. Booster vaccination is defined as one or more doses after a complete primary vaccination schedule, where the first booster dose is at least 7 days before date of blood collection.

Evidence of an infection before the studied breakthrough infection was based on self-report of a positive SARS-CoV-2 test or the presence of N-specific antibodies prior to breakthrough infection.

### Statistical analyses

Log-transformed N-, S1- and RBD-specific responses (BAU/mL values) were modelled using a Gaussian generalized additive model, with the R package mgcv^30^. We modeled the N-, S1- and RBD-specific serological response in individuals without a previous infection as a function of time since positive test in days (in Fig. 1). In addition, for the RBD and S1 responses we expanded this model with an interaction term for time since vaccination and N-seropositivity of the post-infection sample (in Fig. 3). Time since positive test and time since vaccination were included as a tensor product of penalized cubic splines (15 knots), using second order penalties.

Probability of N-seropositivity is modelled using a logistic regression in a generalized additive model as a function of time since positive test (penalized cubic spline, 15 knots, in Fig. 2A). We expand this model separately for categorical variables COVID-19 symptom status (in Fig. 2B, absent/present), variant of infection (Delta/Omicron), vaccination status (partial, full, or booster vaccination), last used vaccine type (Comirnaty, Vaxzevria, Spikevax, or Jcovden), and time since vaccination. Participants only reporting the answer option ‘other symptoms’ were excluded from the analysis into the effect of symptoms. The output of the logistic regression (log-odds) is transformed into a probability of N seropositivity using the inverse logit function, $\mathrm{log}(\frac{{p}_{n+}}{{1- p}_{n+}} )$.

We tested differences in ratios in Delta and Omicron-specific IgG levels between individuals with an Omicron or Delta infection stratified by time since infection with a Wilcoxon test.

### Supplementary Information

Supplementary Information 1.

Supplementary Information 2.

## Data Availability

All antibody data obtained are presented in the manuscript. SARS-CoV-2 variant sequencing data acquired for this study (accession numbers provided in Table S1) are available on https://gisaid.org/.
