# Supplementary material for: Assessment of hybrid population immunity to SARS-CoV-2 following breakthrough infections of distinct SARS-CoV-2 variants by the detection of antibodies to nucleoprotein
Source: Sci Rep. 2023 Oct 26;13:18394. doi: 10.1038/s41598-023-45718-8 (PMC10603038; doi:10.1038/s41598-023-45718-8)
Supplement: Supplementary file 2 — Supplementary Information 2. [file 41598_2023_45718_MOESM2_ESM.pdf]

Assessment of hybrid population immunity to SARS-CoV-2 following breakthrough infections of distinct SARS-CoV-2 variants by the detection of antibodies to Nucleoprotein

Authors:

Gerco den Hartog, Stijn P. Andeweg, Christina E. Hoeve, Gaby Smits, Bettie Voordouw, Dirk Eggink, Mirjam J. Knol, Robert S. van Binnendijk

Supplementary Figures

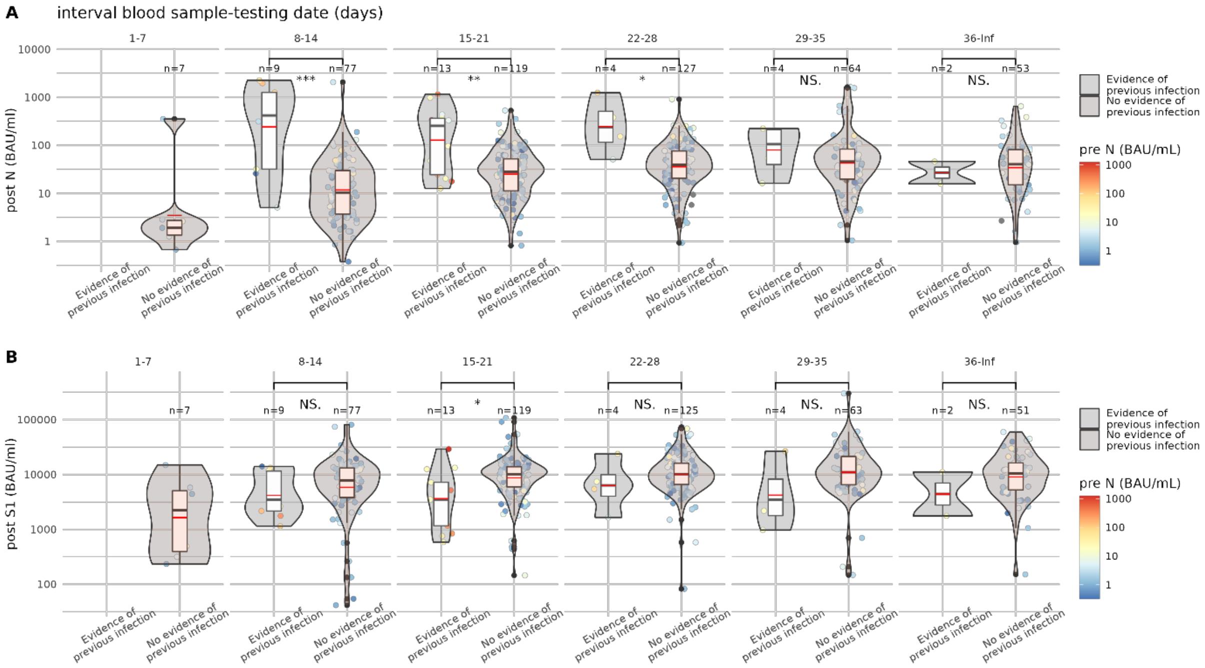

Fig S1. Violin boxplots of the (A) N (BAU/mL) and (B) S1 (BAU/mL) antibody concentrations after infection and dots are colored by the pre infection antibody concentration. *The days since infected are indicated above the panels. Black lines represent the median and red line the geometric mean antibody concentrations. Different subplots indicate time since infection in days. P-values are indicated with *** for < 0.001, ** for < 0.01, * for < 0.05 and not significant (NS).*

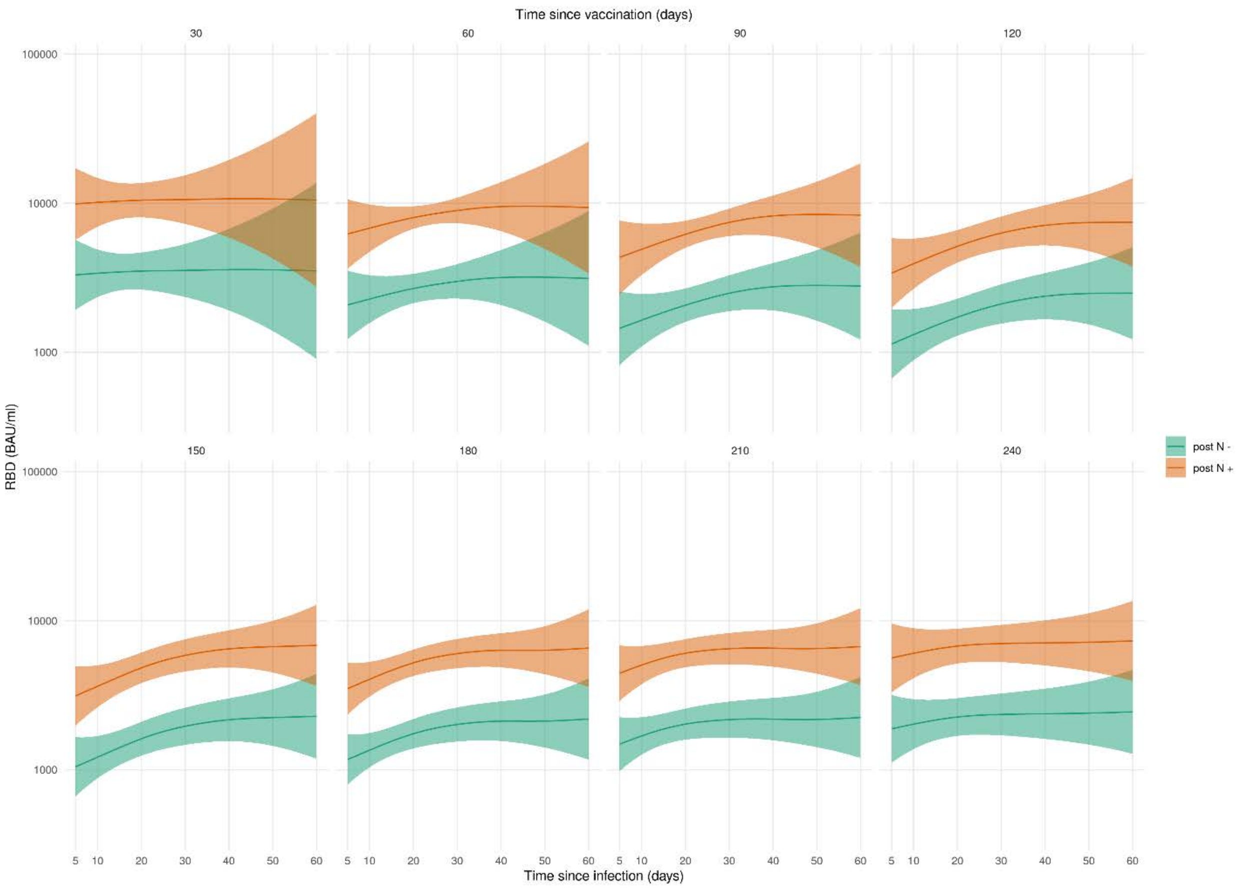

Fig. S2 Estimates of the mean RBD antibody levels as a function of time since infection and vaccination in not previously infected.

Panels show the different time since vaccination (30 days intervals) and x-axis the time since infection. Orange and green indicate the persons with and without N-specific antibodies following breakthrough infection. Shaded areas represent 95% confidence intervals/envelopes.

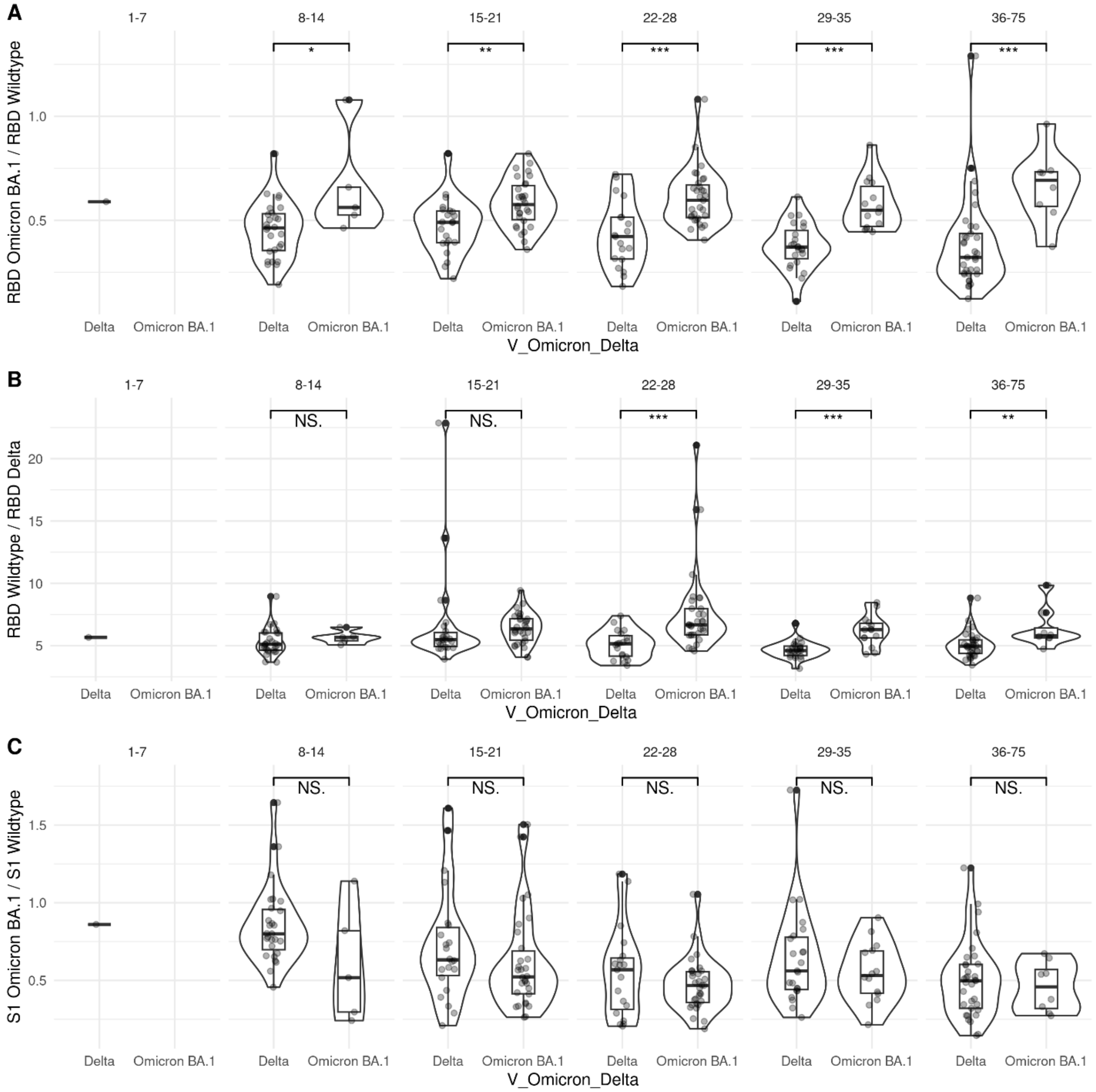

Fig. S3 Ratio of the (A) RBD Omicron BA.1 over RBD Wildtype, (B) RBD Wildtype over RBD Delta, and (C) S1 Omicron BA.1 over S1 Wildtype serological response for Delta and Omicron BA.1 infections. *Different subplots indicate time since infection in days. See Fig S3 for additional antigenic target ratio results. P-value is indicated with *** for < 0.001, ** for < 0.01, * for < 0.05 and not significant (NS).*
